# A Case of Recurrent Urinary Tract Infections With Neurogenic Bladder Due to Spinal Tumors

**DOI:** 10.22037/ijcn.v15i2.27793

**Published:** 2021

**Authors:** Mitra NASERI, Farah ASHRAFZADEH, Mohammad FARAJI RAD, Nourieh SHARIFI

**Affiliations:** 1 Pediatric Nephrology Department, Faculty of Medicine, Mashhad University of Medical Sciences, Mashhad, Iran.; 2 Pediatric Neurology Department, Faculty of Medicine, Mashhad University of Medical Sciences, Mashhad, Iran.; 3 Neurosurgery Department, Faculty of Medicine, Mashhad University of Medical Sciences, Mashhad, Iran.; 4 Pathology Department, Faculty of Medicine, Mashhad University of Medical Sciences, Mashhad, Iran.

**Keywords:** Neurogenic Bladder, Recurrent UTI, Spinal Tumors, Ganglioneuroma

## Abstract

Neuroblastic tumors are the most common extracranial solid tumors in children. They are manifested by different clinical presentations ranging from cord compression symptoms to asymptomatic cases. A 2.5-year girl with a history of vaginal delivery at 39 gestational weeks and low Apgar score presented by repeated episodes of urinary tract infections and progressive paraplegia started at the age of 8 months. Brain MRI and EEG were normal. Voiding cystourethrography revealed grade II vesicoureteral reflux in the left kidney. Lumbar MRI with and without contrast showed a dumbbell shape mass, the hyper signal in T2 -weighted image and low signal in T1 -weighted image, extramedullary, and intramural with mass effect on the cord. Microscopic examination of tissue obtained by surgery reported ganglioneuroma. Our case was interesting because of her presentation, neurogenic bladder associated with repeated episodes of urinary tract infections, and secondary paraplegia. Neurogenic bladder dysfunction is rarely reported in cases with ganglioneuroma.

## Introduction

 Neuroblastic tumors (NT), the most common extracranial solid tumors in childhood, including neuroblastoma, ganglioneuroblastoma (nodular or intermixed), and ganglioneuroma (GN). They originate from the neural crest and may be consisted of immature, undifferentiated to mature or differentiated cells (1). The ganglioneuroblastoma intermixed (GNBI) and GN represent the mature end of this range (2). A wide spectrum of clinical manifestations has been reported in patients with GN ranging from cord compression symptoms to asymptomatic cases (3, 4).

##  Case presentation

 A 2.5-year girl admitted to the nephrology department with fever, leukocyturia in urine analysis, and positive urine culture [*Escherichia coli* with a colony count of > 10^5^ colonies forming unit (CFU)] and diagnosis of acute pyelonephritis. She had a history of an episode of febrile urinary tract infection (UTI) 1.5 years ago. Following the first episode of UTI, kidney –bladder ultrasonography (US) and voiding cystourethrography (VCUG) were done. At that time, the kidney –bladder US was normal, but VCUG revealed grade II vesicoureteral reflux (VUR) in the left kidney. She was the first child of non- consanguine parents, delivered by normal vaginal delivery at 39 gestational weeks with a low Apgar score. She was admitted to the neonatal intensive care unit (NICU) for respiratory distress, hypotonia, poor feeding, and cyanosis after birth for 3 days. Birth weight was 2900 g, birth head circumference was 35 cm, and the mother did not have any problem during pregnancy. Neonatal metabolic screening tests were normal, HTLV-1, and HIV antibodies were negative, purified protein derivative (PPD) test and chest x-ray were also normal. She started creeping at 9 months old, but could not walk until the second year of life with support. There was a history of progressive paraplegia from the age of 8 months. Before that, she was able to move the lower limbs. Developmental skills in the domain of social, language, and fine motor skills were normal. Also, brain magnetic resonance imaging (MRI) and electroencephalography (EEG) performed at the age of 9 months did not reveal abnormal findings. Regarding the history of perinatal asphyxia, the diagnosis was a paraplegic form of cerebral palsy (CP) and physiotherapy was recommended. 

 Kidney–bladder US was repeated after the second hospitalization (age 2.5 years). It showed bilateral mild hydronephrosis and anterior-posterior (AP) diameters of right and left pelvises were 5 and 7 mm, cortical thickness of right kidney (RK) was 6 mm and for left kidney (LK) it was 10 mm. The ultrasound examination of the bladder was normal. Serum creatinine levels were 0.5 mg/dl. Before the second admission, VCUG was repeated based on the recommendation of a nephrologist. It showed grade IV-V VUR in LK. A renal scan (TC ^99^ DMSA scan) was done and revealed a decreased size of LK and significant cortical loss due to multiple scars. The right kidney (RK) had normal cortical uptake. Differential renal function for left and right kidneys were 31% and 69%, respectively. The patient was discharged after cessation of fever with a suitable antibiotic, anticholinergic (oxybutynin), and laxative. She was followed by urine analysis and culture every 2-3 months and recommended to do these tests if fever recurs. During a 17-month follow-up, she had repeated episodes of cystitis and just one episode of febrile UTI, which was managed as an outpatient.

**Figure 1:**
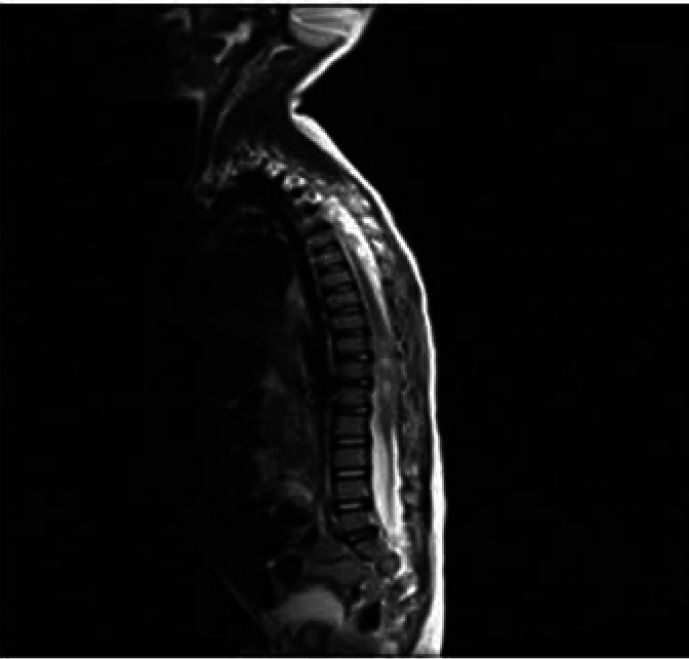
lumbar magnetic resonance imaging (MRI) without contrast that shows a dumbbell shape mass, the hyper signal in T2-weighted image and low signal in T1- weighted image, extramedullary, intramural with mass effect on the cord

**Figure 2:**
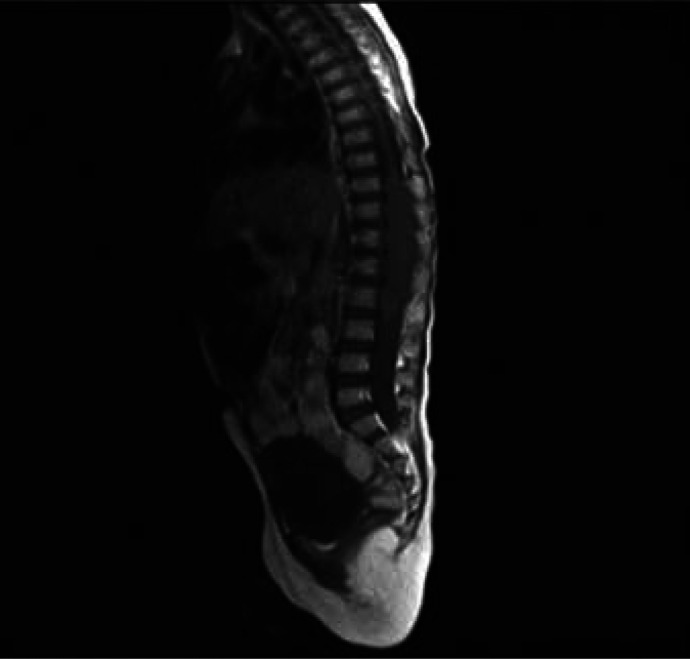
lumbar magnetic resonance imaging (MRI) with contrast, contrast enhancement, spreading along the T10-L2 paravertebral spaces via neural foramen in L2 posterior vertebral body, and scalloping is seen due to bone remodeling

**Figure 3:**
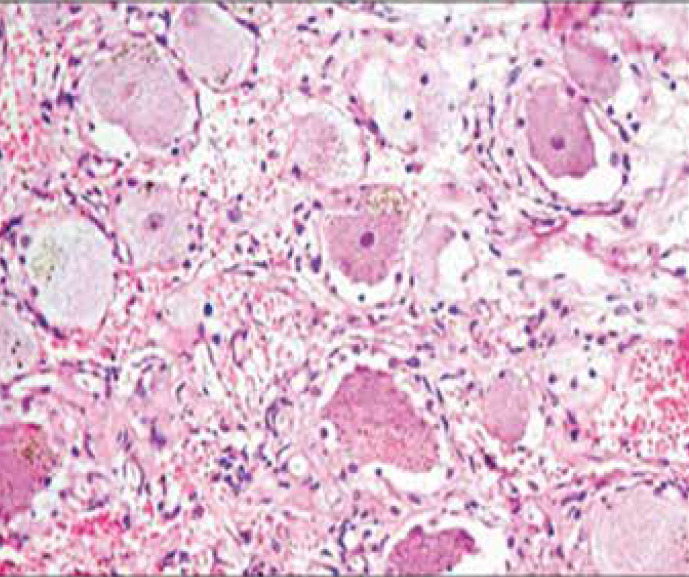
Microscopic view of ganglioneuroma with large ganglion cells and spindle wavy Schwan cells and vascular canals. (H&E.400X).

**Figure 4:**
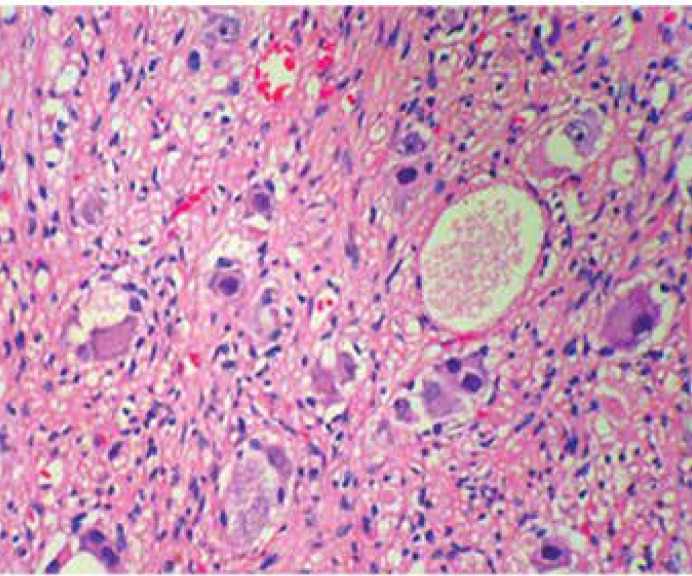
Microscopic view of the ganglioneuroma section shows large nucleotide ganglioneuroma cells and small spindle Schwan cells in the congestive vascularized stroma. (H&E.400X)

 At age 3 years and 7 months (15 months after the first admission), she referred to a pediatric neurologist for more neurologic assessments if needed. She was admitted to the pediatric neurology department due to weakness and hypotonicity in lower extremities accompanied by diminished deep tendon and unresponsive plantar reflexes. She did not cooperate for muscle power determination. Brain MRI was repeated that was normal, lumbar MRI with and without contrast showed a dumbbell shape mass, the hyper signal in T2- weighted image (Fig-1), and low signal in T1-weighted image, extramedullary, intradural with mass effect on the cord. After the injection of contrast media, it showed enhancement, spreading along the T10-L2 paravertebral spaces via neural foramen in the L2 posterior vertebral body, and scalloping was seen due to bone remodeling (Fig-2). Microscopic examination of tissue obtained by surgery reported GN with large ganglion cells and spindle wavy Schwan cells and vascular canals. The microscopic view of the obtained sections showed large nucleated GN and small spindle Schwann cells in congestive vascularized stromal (Figures 3-4).

 After surgery, patients underwent physiotherapy of lower limbs. She was able to elevate slowly both of her lower limbs and could walk with the support of one hand. A urodynamic study was performed 2 months after surgery. Before that, no urodynamic studies were performed because of repeated infections with short intervals. The urodynamic study revealed a bladder capacity of 110 cc and a leak point pressure of 90.8 cm H_2_o_._ The estimated bladder volume according to the age (3 years and 9 months) was 177 cc. The bladder capacity of patients was 62% of the average aged-matched capacity and indicated small bladder capacity with high intravesical pressure. Clean intermittent catheterization (CIC) was recommended, but parents did not accept to do it. Unfortunately, the spinal surgery associate with medical treatment for neurogenic bladder dysfunction did not improve the recurrence of UTIs. Ten months after surgery, the serum creatinine level was 0.5 mg/dl. Voiding cystourethrogram was obtained and showed resolution of VUR. The patient had a sensation of bladder and bowel filling, and bowel but not bladder control. 

 Despite neurosurgeon recommendation, patients lost the follow-up for 12 months. One year after surgery, she was examined by a neurosurgeon, and spinal MRI was repeated. It showed the recurrence of the tumor. Focuses of abnormal signals on the cord and thecal sac space, especially on the right side in the level of T12 with compression of cord and canal stenosis were reported. 

## Discussion

 GN generally has a benign course and almost always is treated by surgery (5). Clinical manifestation and biologic behavior of primary GN was assessed in 49 patients (6). The median age at diagnosis was 79 months, with equal distribution among females and males (M/F ratio of 1.13:1). Tumors were localized in the thoracic cavity (41.5%) and abdominal and non-adrenal regions (37.5%). An increase in plasma and/or urine levels of catecholamines were seen (39% of patients) and slight immaturity of ganglion cells (93%) were defined, as well. In a 25-month follow-up, the tumors had benign behaviors with no progression or recurrence.

 Tumor resection without any cytotoxic treatment was recommended as the main treatment, and the final outcome was usually excellent (7). In children aged > 10 years, localization of tumors mainly is in the mediastinum, and association of the tumor with neuroblastoma has been found in rare cases (8). Additionally, the association of neurofibromatosis with diffuse intestinal GN has been reported (9, 10). By searching and reviewing the literature, we just found a case of GN with presentations similar to our case (11). Ljung et al. (11) in 1984 reported a 6-year-old girl with neurogenic bladder dysfunction, gait disorder, and progressive foot deformities. The etiology of neurogenic bladder and gait disorder, in this case, was intraspinal GN. No details about the urinary presentation of this patient were available. Our case was interesting because of the presentations (paraplegia and recurrent UTI). In our case, because of the low Apgar score at delivery, cerebral palsy due to asphyxia was the first diagnosis. Abnormal findings in physical examination that suggested a lesion in lower neuron motor (hypotonicity in lower limbs accompanied by diminished deep tendon reflexes and unresponsive plantar reflexes) accounted for the suspension to presence of a spinal cord lesion. 

##  Conclusion

Although spinal tumors are a rare reason for neurogenic bladder in children, they should be considered if there are unusual neurologic findings in physical examination. In each patient who referred with a diagnosis of cerebral palsy, if there are any incredulity during the physical examination, more evaluations should be considered.
